# Pituitary neuroendocrine tumor: evaluation with super resolution deep learning reconstruction: Research

**DOI:** 10.1007/s00234-025-03819-3

**Published:** 2025-10-21

**Authors:** Koichiro Yasaka, Akira Katayama, Naoya Sakamoto, Yuko Sato, Yusuke Asari, Jun Kanzawa, Yuki Sonoda, Yuichi Suzuki, Shiori Amemiya, Shigeru Kiryu, Osamu Abe

**Affiliations:** 1 The University of Tokyo, Tokyo, Japan; 2 International University of Health and Welfare, Ōtawara, Japan

**Keywords:** Pituitary neuroendocrine tumor, Pituitary stalk, Magnetic resonance imaging, Deep learning reconstruction, Super-resolution

## Abstract

**Purpose:**

To evaluate the impact of super-resolution deep learning reconstruction (SR-DLR) algorithm on the evaluations of pituitary neuroendocrine tumor (PitNET) and on the image quality of pituitary MRI compared to conventional images with zero-filling interpolation (ZIP) technique.

**Methods:**

This retrospective study included 29 patients with PitNET who underwent pituitary MRI imaging. T2-weighted coronal images were reconstructed with SR-DLR and ZIP. Three readers assessed the images in terms of pituitary stalk deviation, noise, sharpness, depiction of PitNET, and diagnostic acceptability. A radiologist placed circular or ovoid regions of interest (ROIs) on the lateral ventricle and the tumor, and signal-to-noise ratio (SNR) and contrast-to-noise ratio were calculated. The radiologist also placed a linear ROI crossing the septum pellucidum perpendicularly. From the signal intensity profile along this ROI, edge rise slope (ERS) and full width at half maximum (FWHM) were calculated.

**Results:**

Inter-reader agreement in the evaluations of pituitary stalk deviation in SR-DLR (0.518) tended to be superior to that in ZIP (0.405). Scores in the qualitative image analyses in SR-DLR were significantly better than those in ZIP for all evaluation items (*p* < 0.001). SNR and CNR in SR-DLR were significantly higher compared to ZIP (*p* < 0.001). Results of ERS (5433/2177 in SR-DLR/ZIP) and FWHM (0.67/1.27 mm in SR-DLR/ZIP) indicated significantly enhanced spatial resolution in SR-DLR compared to ZIP.

**Conclusion:**

SR-DLR tended to enhance inter-reader agreement in the evaluations of pituitary stalk deviation and significantly improved quality of pituitary MRI images compared to conventional ZIP images.

## Introduction

Pituitary neuroendocrine tumor (PitNET) accounts for approximately 15% of all intracranial neoplasms [1]. PitNETs can be categorized into microadenomas (with dimensions equal to or less than 10 mm) and macroadenomas (exceeding 10 mm). PitNETs can manifest with hormonal symptoms, while macroadenomas may also cause cranial nerve symptoms, such as those resulting from cavernous sinus space invasion. MRI plays a crucial role in evaluating the presence, location, size, local compressive mass effects, and invasiveness of PitNETs [2].

Given that PitNETs originate from the pituitary gland, a small structure, MRI imaging of PitNET requires high-resolution images with reduced reconstruction diameter and thinner slice thickness. In addition to high-resolution imaging, dynamic studies aid in detecting microadenomas by enhancing the contrast between the PitNET and normal pituitary gland [3]. The use of an ancillary imaging feature, such as pituitary stalk deviation [4], facilitates the localization of microadenomas. However, increasing spatial resolution is associated with employing smaller voxel size, which can result in a decrease in the signal from each voxel. Consequently, high-resolution pituitary MRI images may exhibit a lower signal-to-noise ratio (SNR)–in other words, increased image noise. Contrast materials pose challenges in administration to patients with renal impairment or allergies.

Since mid-2010s, applications of deep learning have garnered significant attention in the radiology field [5, 6]. Deep learning has proven beneficial in various aspects, including diagnostic imaging [7, 8], radiology report classification [9, 10], and image processing [11–13]. Recently, super-resolution deep learning reconstruction (SR-DLR) algorithms have emerged, capable of enhancing the spatial resolution of MRI images without compromising SNR [14–16]. Successful applications of SR-DLR have been reported in evaluating diseases associated with small structures, such as neurovascular compression [17], cerebral microbleeds [18], cervical neuroforaminal stenosis [19], cauda equina in lumbar spinal stenosis [20]. Given the accumulating evidence, it is plausible that SR-DLR can serve as a valuable tool to enhance the quality of unenhanced pituitary MRI images.

The aim of this study was to evaluate the impact of SR-DLR on the quality of unenhanced pituitary MRI images and the evaluations of PitNET by comparing them with conventional images.

## Materials and methods

This retrospective study was approved by our Institutional Review Board, which waived the requirement for obtaining written informed consent from patients.

### Patients

Patients with PitNET who underwent pituitary MRI at an MRI unit from July 2024 to February 2025 were included in this study. Those who were in the post operative state (*n* = 4) and those who underwent gamma knife radiosurgery (*n* = 1) were excluded. Additionally, for patients who underwent MRI more than once during the study period, any examinations after the first MRI were excluded (*n* = 2).

## MRI imaging

Coronal pituitary T2-weighted imaging was performed with a 3 T MRI unit (Vantage Centurian, Canon Medical Systems). MRI imaging parameters were as follows: repetition time, 4000 ms; echo time, 81 ms; flip angle, 90 degrees; echo train length, 16; number of phase encoding steps, 450; acquisition matrix, 272/224; and number of averages, 1.0. From the source data, images were reconstructed using SR-DLR (Precise IQ Engine, commercially available from Canon Medical Systems) and conventional method with zero filling interpolation (ZIP). The ZIP factors for SR-DLR and ZIP were 3 and 2, respectively. Row/columns/pixel spacing in SR-DLR and ZIP were 816/816/0.1716 mm and 544/544/0.2574 mm, respectively. Reconstruction diameter (140 mm), slice thickness (3 mm), and space between slices (3.3 mm) were identical between SR-DLR and ZIP.

## Concept of SR-DLR

We employed a commercially available SR-DLR algorithm that comprises three steps to enhance spatial resolution without compromising SNR. This algorithm involves the following steps:

1. Image processing: Imaging data are initially processed using a convolutional neural network trained to reduce image noise.
2. ZIP: After images are processed with fast Fourier transform, ZIP of the k-space is conducted with ZIP factor of 3. ZIP is a technique that enhances spatial resolution in MRI images. However, it is known that using a higher ZIP factor can result in ringing artifacts.
3. Artifact reduction: After data are processed with inverse fast Fourier transform, the second convolutional neural network, trained to reduce such artifacts, is applied. This step ensures that the enhanced spatial resolution is achieved without introducing ringing artifacts.

## Qualitative image analysis

Three readers (Reader 1, 2, and 3 with diagnostic imaging experience of 9, 7, and 4 years, respectively) participated in the qualitative image analyses. Prior to the analyses, a radiologist (Radiologist A, with 15 years of diagnostic imaging experience) randomized all image datasets. The location of the PitNET (Radiologist A reviewed coronal T2-weighted images and, if available, contrast-enhanced MRI images) was communicated to the readers. Readers were blinded to other patient background information. Using Image J (https://imagej.net/ij/), the readers independently evaluated the images based on the following evaluation items:

-  Deviation of pituitary stalk (right, middle, or left).
- Invasion of cavernous sinus space according to the Knosp classification algorithm [21, 22] (grade 0 = not encroach on the cavernous sinus space, grade 1 = the medial tangent passage, grade 2 = extension beyond the intercarotid line, grade 3 = lateral extension to the lateral tangent of the intracavernous and supracavernous internal carotid arteries into the superior or inferiorcavernous sinus compartment, grade 4 = total encasement of the intracavernous carotid artery).
- Noise (4 = no or minimal, 3 = mild, 2 = moderate, 1 = severe interfering with diagnosis).
- Sharpness (4 = sharp, 3 = slightly blurred in a small region, 2 = blurred for some regions, 1 = blurred overall).
- Artifact (4 = no or minimal, 3 = mild, 2 = moderate, 1 = severe interfering with diagnosis).
- Depiction of PitNET (4 = clear, 3 = slightly unclear, 2 = moderately unclear, 1 = unclear overall).
- Diagnostic acceptability (4 = optimal, 3 = slightly degraded quality not interfering with diagnosis, 2 = moderately degraded quality, 1 = unacceptable).
- Diagnostic acceptability (4 = optimal, 3 = slightly degraded quality not interfering with diagnosis, 2 = moderately degraded quality, 1 = unacceptable).

## Quantitative image quality analysis

**Fig. 1:**
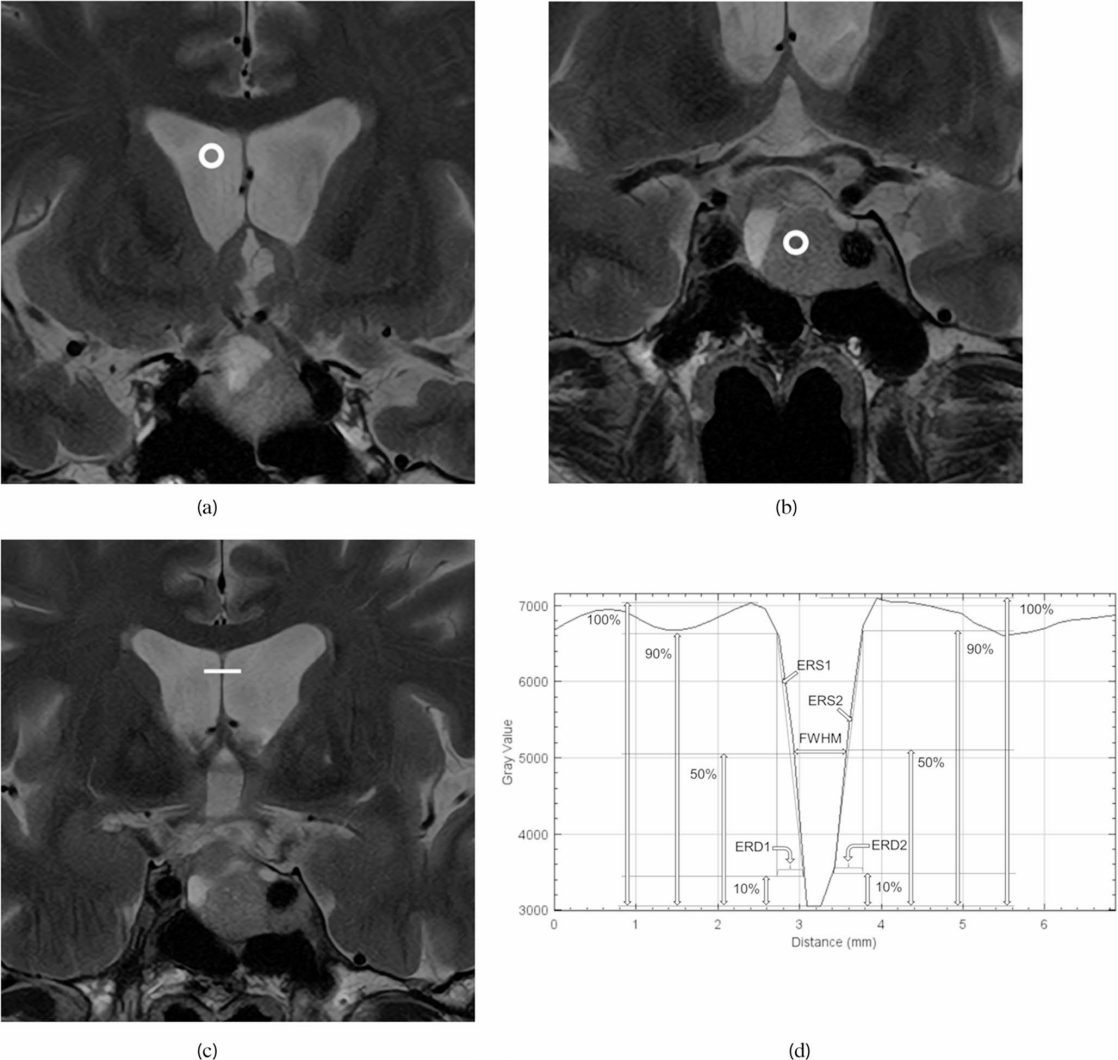
Circular or ovoid regions of interest were placed on the right lateral ventricle (**a**) and pituitary neuroendocrine tumor (**b**). A linear region of interest crossing the septum pellucidum perpendicularly was also placed. From the signal intensity profile along this region of interest, the full width at half maximum (FWHM), edge rise distance (ERD, average of ERD1 and ERD2), and edge rise slope (ERS, average of ERS1 and ERS2) were calculated

Radiologist A placed circular or ovoid regions of interest (ROIs) on the right lateral ventricle with a diameter of approximately 5 mm (Fig. 1a) and on homogeneous region of the tumor with a diameter of up to 5 mm (adjusted to tumor’s size) (Fig. 1b). Subsequently, the mean and standard deviation of the signal intensity within the ROIs were recorded. Then, the SNR for the right lateral ventricle (SNR_CSF_) and the tumor (SNR_TUMOR_) and contrast-to-noise ratio (CNR) between the right lateral ventricle and the tumor were calculated using the following formula:

- SNR_CSF_ = (mean signal intensity in the lateral ventricle)/(standard deviation of the signal intensity in the lateral ventricle).
- SNR_TUMOR_ = (mean signal intensity in the tumor)/(standard deviation of the signal intensity in the tumor).
- CNR = (mean signal intensity in the lateral ventricle - mean signal intensity in the tumor)/√(((standard deviation of the signal intensity in the lateral ventricle)^2^ + (standard deviation of the signal intensity in the tumor)^2^)/2).

Radiologist A also placed a linear ROI that intersects the septum pellucidum perpendicularly (Fig. 1c). From the signal intensity profile along the ROI, the full width at half maximum (FWHM), edge rise distance (ERD), and edge rise slope (ERS) were calculated (Fig. 1d).

### Statistical analysis

Statistical analyses were conducted using R version 4.1.2 (https://www.r-project.org/). Inter-reader agreements in the evaluations of pituitary stalk deviation and cavernous sinus space invasion were assessed using linearly weighted kappa analyses. Results for the qualitative and quantitative image analyses were compared between SR-DLR and ZIP with Wilcoxon signed rank test and paired *t*-test, respectively. A *p*-value less than 0.050 was deemed statistically significant. In this study, we compared two groups and did not perform any multiple comparisons.

## Results

### Patients

In this study, 29 patients (mean age, 52.4 ± 15.7 years; 16 males and 13 females) with the PitNET were included. The median size of PitNETs was 12 mm (interquartile range, 8–18 mm). There were 11 microadenomas and 18 macroadenomas.

### Pituitary stalk deviation and cavernous sinus space invasion

**Fig. 2:**
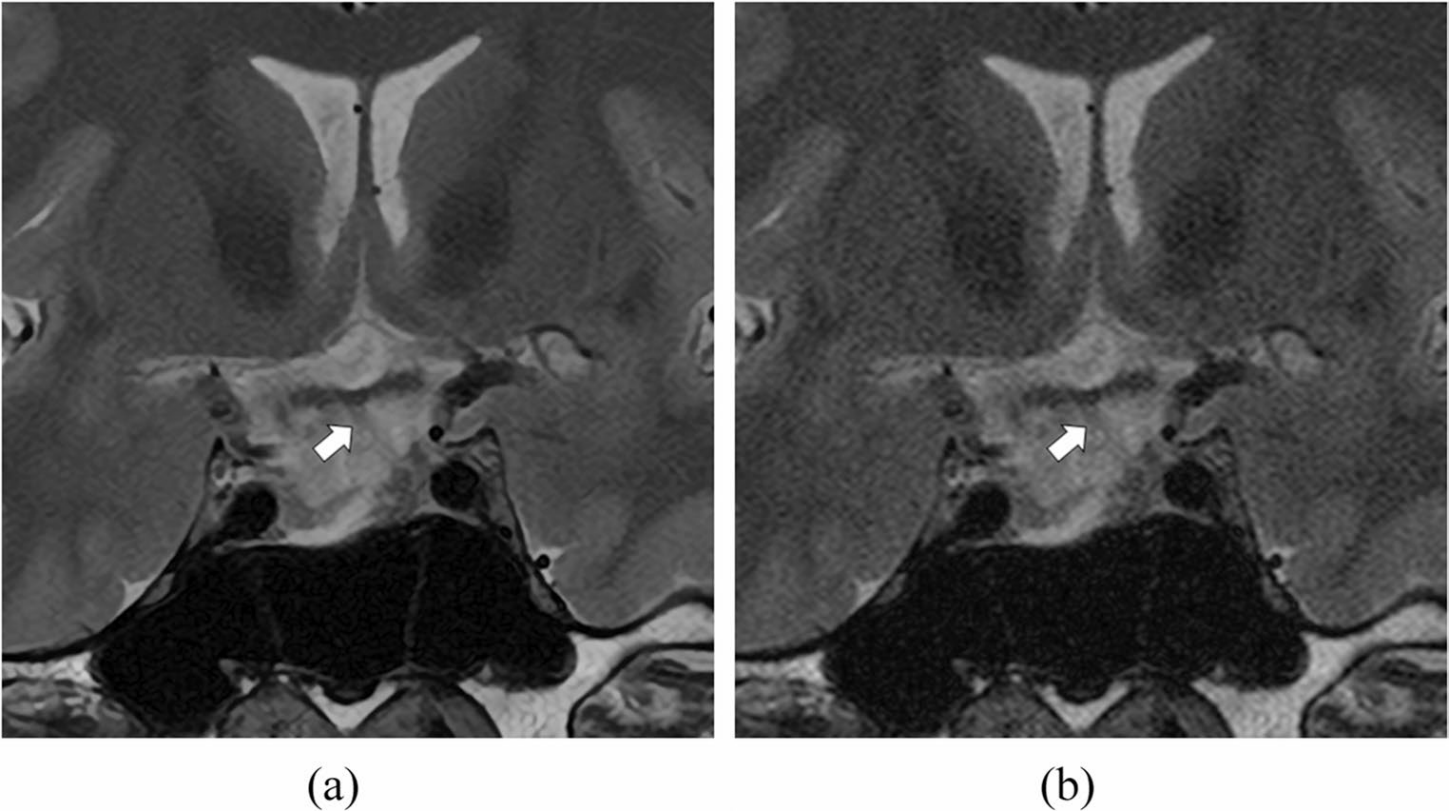
T2-weighted MRI images of a 33-year-old male patient with super-resolution deep learning reconstruction (**a**) and conventional zero-filling interpolation (**b**). The pituitary stalk (arrows) was rated as deviated to left/left/middle in (**a**) and middle/left/right in (**b**) by readers 1/2/3

Each reader’s scores for pituitary stalk deviation and cavernous sinus space invasion are provided in Table 1. Inter-reader agreement in the evaluations of pituitary stalk deviation and cavernous sinus space invasion is shown in Table 2. The average inter-reader agreement in the evaluations of pituitary stalk deviation with SR-DLR was 0.518, which tended to be superior to that with ZIP (0.405) (Fig. 2). Furthermore, inter-reader agreement for pituitary stalk deviation in SR-DLR was improved compared to ZIP between all pairs of readers. The average inter-reader agreement in the evaluations of cavernous sinus space invasion with SR-DLR (0.621) was almost equivalent to that with ZIP (0.638).

**Table 1:** Scores for pituitary neuroendocrine tumor evaluations

		SR-DLR			ZIP		
		Reader 1	Reader 2	Reader 3	Reader 1	Reader 2	Reader 3
Pituitary stalk deviation	Right	6	5	5	5	5	5
	Middle	20	19	17	21	20	24
	Left	3	5	7	3	4	0
Cavernous sinus space invasion	Grade 0	6	9	10	8	10	13
	Grade 1	9	6	7	6	8	5
	Grade 2	5	7	7	7	5	5
	Grade 3	4	4	1	6	3	4
	Grade 4	5	3	4	2	3	2

SR-DLR, super-resolution deep learning reconstruction; DLR, deep learning reconstruction

**Table 2:** Inter-reader agreement for the pituitary neuroendocrine tumor evaluations

	Reconstruction	Average	Reader 1 vs. 2	Reader 2 vs. 3	Reader 3 vs. 1
Pituitary stalk deviation	SR-DLR	0.518	0.816	0.364	0.377
	ZIP	0.405	0.754	0.270	0.190
Knosp classification	SR-DLR	0.621	0.617	0.581	0.665
	ZIP	0.638	0.646	0.713	0.557

SR-DLR, super-resolution deep learning reconstruction; DLR, deep learning reconstruction

### Qualitative image quality analysis

**Fig. 3:**
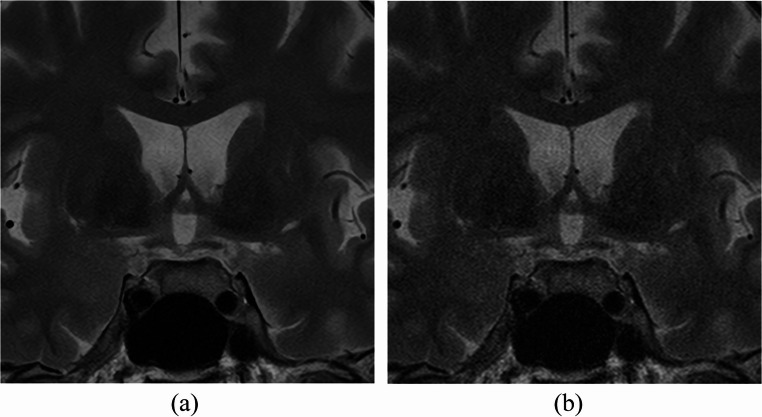
T2-weighted MRI images of a 58-year-old male patient with super-resolution deep learning reconstruction (**a**) and conventional zero-filling interpolation (**b**). Scores for sharpness were 4 (sharp)/4/4 in (**a**) and 1 (blurred overall)/1/2 (blurred for some regions) in (**b**) by readers 1/2/3

**Fig. 4:**
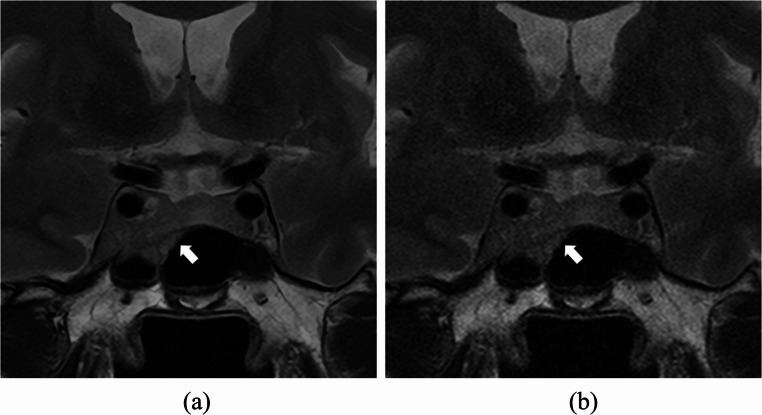
T2-weighted MRI images of a 65-year-old female patient with super-resolution deep learning reconstruction (**a**) and conventional zero-filling interpolation (**b**). Scores for depiction of pituitary neuroendocrine tumor (arrows, 36 mm in diameter) were 3 (slightly unclear)/4 (clear)/4 in (**a**) and 1 (unclear overall)/2 (moderately unclear)/2 in (**b**) by readers 1/2/3

**Fig. 5:**
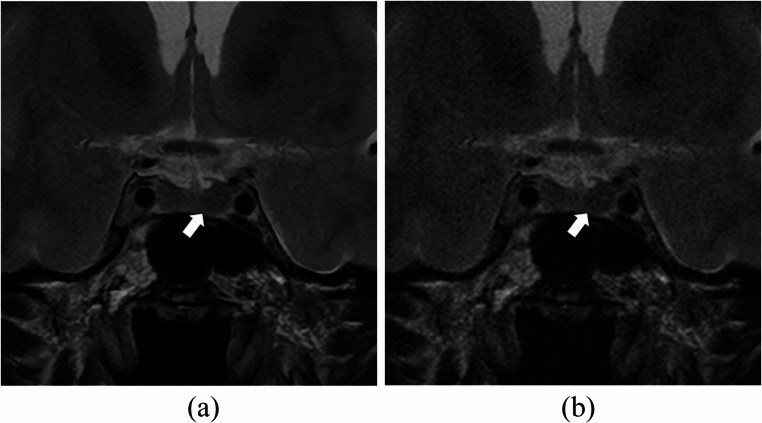
T2-weighted MRI images of a 38-year-old female patient with super-resolution deep learning reconstruction (**a**) and conventional zero-filling interpolation (**b**). Scores for depiction of pituitary neuroendocrine tumor (arrows, 6 mm in diameter) were 4 (clear)/4/4 in (**a**) and 3 (slightly unclear)/3/2 (moderately unclear) in (**b**) by readers 1/2/3

Results of qualitative image quality analyses are provided in Table 3. Three readers agreed that the quality of SR-DLR images is significantly superior to that of ZIP in terms of all evaluation items (noise, sharpness [Figure 3], artifact, depiction of PitNET [Figs. 4 and 5], diagnostic acceptability) (*p* < 0.001 for all).

**Table 3:** Results for qualitative image quality analysis

	Reader	SR-DLR	ZIP	*P*-value
Noise	1	15/9/4/1	0/1/24/4	< 0.001*
	2	15/14/0/0	0/1/24/4	< 0.001*
	3	8/21/0/0	0/0/29/0	< 0.001*
Sharpness	1	12/11/5/1	1/4/13/11	< 0.001*
	2	18/11/0/0	1/14/11/3	< 0.001*
	3	18/11/0/0	0/0/29/0	< 0.001*
Artifact	1	18/7/3/1	4/16/5/4	< 0.001*
	2	7/21/1/0	0/18/10/1	< 0.001*
	3	11/18/0/0	0/1/28/0	< 0.001*
Depiction of PitNET	1	10/11/7/1	1/8/12/8	< 0.001*
	2	15/11/3/0	2/18/9/0	< 0.001*
	3	15/13/1/0	0/1/24/4	< 0.001*
Diagnostic acceptability	1	11/10/7/1	1/8/10/10	< 0.001*
	2	16/13/0/0	0/4/24/1	< 0.001*
	3	15/14/0/0	0/1/24/4	< 0.001*

SR-DLR, super-resolution deep learning reconstruction; DLR, deep learning reconstruction; PitNET, pituitary neuroendocrine tumor

The numbers of patients for each score (4/3/2/1) are presented. Comparisons are performed with Wilcoxon signed rank test. *, statistically significant difference (*p* < 0.050)

### Quantitative image quality analysis

In the SR-DLR, SNR_CSF_ was 33.3 ± 7.6, which was significantly higher than that in the ZIP (20.0 ± 3.0) (*p* < 0.001). Similarly, the SNR_TUMOR_ in the SR-DLR was 11.4 ± 5.7, which was also superior to that in the ZIP (7.8 ± 3.2) (*p* < 0.001). CNR of the SR-DLR was 12.7 ± 5.7, which was significantly higher than that of the ZIP (8.5 ± 3.9) (*p* < 0.001).

In the SR-DLR, FWHM was 0.67 ± 0.19 mm, which was significantly smaller than that in the ZIP (1.27 ± 0.72 mm) (*p* < 0.001). ERS in the SR-DLR was 5433 ± 2959, which was significantly higher than that in the ZIP (2177 ± 1395) (*p* < 0.001). ERD in the SR-DLR and the ZIP was 1.22 ± 0.57 mm and 1.39 ± 0.72 mm, respectively, and there was no statistically significant difference between them (*p* = 0.171).

## Discussion

Pituitary MRI necessitates high spatial resolution images, which inevitably lead to increased noise. In this study, we discovered that the SR-DLR algorithm successfully achieved both spatial resolution enhancement and image noise reduction, resulting in significantly improved visualization of PitNET. Inter-reader agreement in the evaluations of pituitary stalk deviation also tended to be improved with SR-DLR images compared to conventional ZIP images.

Pituitary stalk deviation is an ancillary imaging feature of PitNET, which aids in predicting its location within the sella turcica, particularly for microadenomas. From this study, we observed that inter-reader agreement in the evaluations of pituitary stalk deviation with SR-DLR tends to improve compared to ZIP. Conversely, there was no remarkable difference in the inter-reader agreement in the evaluations of cavernous sinus space invasion between SR-DLR and ZIP. This may be attributed to the fact that the pituitary stalk is a small structure, with its diameter reported as 1.91 mm at its pituitary insertion [23]. Enhanced spatial resolution achieved with SR-DLR would have positively impacted the evaluations of pituitary stalk deviation.

The pituitary gland is a small structure and pituitary MR imaging are often obtained with smaller reconstruction diameters and thinner slice thicknesses. However, MRI images with higher spatial resolution are generally associated with reduced SNR, as the MRI signal from each voxel diminishes. In this study, we confirmed that the SR-DLR algorithm achieved enhanced spatial resolution without compromising SNR compared to conventional ZIP images in both qualitative and quantitative image quality analyses for pituitary MRI. Specifically, SNR and CNR in quantitative image analyses and scores for image noise in qualitative image analyses in SR-DLR were significantly higher than those in ZIP images. Furthermore, results for FWHM and ERS in the quantitative analyses and scores for sharpness in the qualitative analyses indicated improved spatial resolution in SR-DLR images compared to those in ZIP images.

In this study, the SR-DLR algorithm employed utilizes the ZIP process with a ZIP factor of 3. The use of such high ZIP factors is known to be associated with the generation of ringing artifacts. However, artifacts in SR-DLR were rated as significantly reduced compared to ZIP images. This suggests that the second convolutional neural network within the SR-DLR algorithm successfully achieved artifact reduction in pituitary MRI images and that it can be safely applied to pituitary assessment.

The combination of enhanced spatial resolution, reduced image noise (or improved SNR and CNR), and reduced artifacts has resulted in significantly improved depiction of PitNET and diagnostic acceptability in SR-DLR compared to ZIP. Although some previous articles have reported that reconstruction algorithms based on deep learning strategies are beneficial in enhancing the quality of contrast-enhanced pituitary MRI images [24, 25], those results indicate that the pituitary MRI images are better reconstructed with SR-DLR rather than ZIP, also for coronal pituitary T2-weighted images in the clinical practice. Our findings would be advantageous for patients with renal function impairment or a history of allergy to gadolinium-based contrast materials. Additionally, since gadolinium-based contrast materials may not be necessary for postoperative imaging in many cases of macroadenoma [26], our results could also be beneficial for such patients. While we did not measure the reconstruction time for SR-DLR, there was no large difference in the reconstruction time between SR-DLR and conventional ZIP images.

There are some limitations with this study. Firstly, the number of patients included in this study was relatively small. However, because promising results were obtained with our study, future prospective study enrolling a larger number of participants is warranted. Secondly, there is a risk that readers may be aware of the reconstruction algorithms in the qualitative image analyses. However, we made efforts to randomize the datasets prior to the qualitative image analyses and ensure that the readers were unaware of background information. Third, not all patients underwent surgery after MRI examination, so correlation between image findings and surgical or pathological findings was not performed. Fourth, microadenomas typically are evaluated using dynamic contrast-enhanced MRI. Future research focusing on the assessment of microadenomas with this technique would be necessary. Finally, while similar algorithms are available from other vendors, the detailed mechanisms differ across them. Therefore, our study results would not be directly applicable to those other vendors’ algorithms.

In conclusion, pituitary MRI images reconstructed using SR-DLR exhibited enhanced spatial resolution, improved SNR, and reduced artifacts, leading to significantly improved visualization of PitNET and enhanced diagnostic acceptability. Additionally, inter-reader agreement in the evaluations of pituitary stalk deviation in SR-DLR was generally superior to that in ZIP images. Consequently, it is recommended to reconstruct pituitary T2-weighted coronal MRI images using SR-DLR rather than conventional ZIP in routine clinical practic

## Data Availability

No datasets were generated or analysed during the current study.
